# Knowledge about Obstetric Danger Signs and Associated Factors among Mothers in Tsegedie District, Tigray Region, Ethiopia 2013: Community Based Cross-Sectional Study

**DOI:** 10.1371/journal.pone.0083459

**Published:** 2014-02-06

**Authors:** Desta Hailu, Hailemariam Berhe

**Affiliations:** 1 Department of Nursing, College of Medicine and Health Sciences, Arba Minch University, Arba Minch, Ethiopia; 2 Department of Nursing, College of Medicine and Health Sciences, Mekelle University, Mekelle, Ethiopia; University of Alabama at Birmingham, United States of America

## Abstract

**Background:**

In many developing countries including Ethiopia, maternal morbidity and mortality still pose a substantial burden and thus progress towards the fifth Millennium Development Goal (MDG) remains slow. Raising awareness of women about the danger signs of pregnancy and childbirth is the first essential step in accepting appropriate and timely referral to obstetric care. However, in Ethiopia little is known about the knowledge level of mothers about obstetric danger signs. The objective of this study was to assess the status of knowledge of danger signs of pregnancy and childbirth among mothers who gave birth in the past two years prior to the survey in Tsegedie district, Tigray regional state, Ethiopia.

**Methods:**

A Community based cross-sectional study was conducted from November 20, 2012 to June 30, 2013 on a randomly selected sample of 485 women who had at least one delivery in the past two years. Multistage sampling technique was employed to select the study participants. A pre-tested structured questionnaire was used to collect quantitative data. Focus group discussion and in-depth interviews were utilized to supplement the Quantitative data. Bivariate and multivariate data analysis was performed using SPSS version 17.0 software.

**Result:**

Four hundred eighty five mothers participated in the study making a response rate of 100%. Vaginal bleeding was the most commonly mentioned danger signs of pregnancy (49.1%) and childbirth (52.8%). Two hundred eighty five (58.8%) and 299 (61.6%) of respondents mentioned at least two danger signs of pregnancy and childbirth respectively. One hundred seventy (35.1%) and 154 (31.8%) of respondents didn't know any danger signs of pregnancy and childbirth respectively. Educational status of the mother, place of delivery and having functional radio were found to be independent predictors of knowledge of women about the danger signs of pregnancy and childbirth.

**Conclusion:**

Educational status of the mother, place of delivery and having functional radio were independently associated with knowledge of women about obstetric danger signs. Thus, provision of information, education and communication targeting women, family and the general community on danger signs of pregnancy and childbirth and associated factors was recommended.

## Introduction

In many developing countries including Ethiopia, maternal mortality still remains a substantial burden and hence progress towards the fifth Millennium Development Goal (MDG) has been particularly slow [1]. Childbearing is experienced not as the joyful event as it should be. Each year, approximately 287,000 women die from complications related to pregnancy and childbirth, with 99% of these deaths occurring in developing countries. Maternal mortality has shown to have large discrepancies between developed and developing countries. Maternal mortality ratio (MMR) in developing regions is 15 times (240/100,000 live births) higher than in developed regions (16/100,000live births). Sub-Saharan Africa had the highest MMR at 500 maternal deaths per 100,000 live births [2]. In Ethiopia, levels of maternal mortality and morbidity are among the highest in the world. According to Ethiopian demography and health survey (EDHS) 2011report,MMR was 676 per 100,000 live births [3], which is slightly higher than EDHS 2005 report (673 per 100,000 live births) [4].

A mother's death has profoundly bad consequences for her family, particularly for children left without care taker. In less developed countries, if the mother dies, the risk of death for her children under age 5 can increase by as much as 50 percent. Besides to this, because these women are stricken during their most productive years, their deaths have a profound impact on the society and on the economies of their nations at large [5].

Furthermore, for every maternal death, many more women are suffering from injuries, infections and disabilities related to pregnancy and childbirth [5]. According to world health organization (WHO), in the developing world Over 30 million women suffer each year from serious obstetric complications as a result of inadequate or inappropriate care during pregnancy, delivery and the first few critical hours after birth [6].

Maternal deaths have both direct and indirect causes. Around 80% of maternal deaths worldwide is brought about by direct obstetric complications such as hemorrhage, infection, obstructed and prolonged labor, unsafe abortion and hypertensive disorders of pregnancy. Indirect causes such as malaria, diabetes, hepatitis, anemia and other cardiovascular disorders which are aggravated by pregnancy can also lead to maternal death [6].

The danger signs are not the actual obstetric complications, but symptoms that are easily identified by non-clinical personnel. They are mainly classified into three; the commonest/key danger signs during pregnancy include severe vaginal bleeding, swollen hands/face and blurred vision. Major danger signs during labor and childbirth include Severe vaginal bleeding, Prolonged labor (>12 hours), Convulsions and retained placenta. Major danger signs during the postpartum period include severe vaginal bleeding, foul-smelling vaginal discharge, and fever [7].

Maternal morbidity and mortality could be prevented significantly if women and their families recognize obstetric danger signs and promptly seek health care service during labor, delivery and early postpartum period under the supervision of skilled delivery attendant(SBA). Evidence suggests that raising awareness of women about obstetric danger signs would improve early detection of problems and reduces the delay in deciding to seek obstetric care [8]. It is the essential first step in the appropriate and timely referral to essential obstetric care. Similarly, because most babies are born at home or are discharged from the hospital in the first 24 hours, increasing community awareness of the danger signs of newborn complications is of critical importance for improving newborn survival [7]. Thus, this has been identified as one of the key strategies for improving maternal and child health [8]. However, like in many developing countries, awareness of women about obstetric danger signs remains low in Ethiopia [9].

The Federal Ministry of Health (FMOH), reproductive health department and Health Bureaus of respective regions have made concerted effort to promote awareness of mothers about obstetric danger signs and achieve Millennium Development Goal 5 on a date. They have been Appling multi-pronged approaches at local and national levels to improve access to health care information throughout the country including activities such as training of health care providers and health extension workers, organizing civil societies, supporting women to women's associations (such as Health development army, women's networks etc.), increasing access to health facilities and allocating health resources more equitably among rural and urban areas [10].

Although women's awareness about obstetric danger signs has substantial importance for improving maternal and child health, little is known about the current knowledge and influencing factors in the study area. This study therefore aims to fill this gap by assessing the current level of knowledge and determinant factors among urban and rural women who gave birth in the past two years prior to this survey in Tigray region, Ethiopia.

## Materials and Methods

### Study setting and period

This study was conducted from October 20, 2012 to June 19, 2013 in Tsegedie district. Tsegedie is one of the 48 districts found in the western zone of the Tigray Regional State. The administrative town of this district, Ketema Nigus is located 876 Kms to North West of Addis Ababa. As projected from 2007 Ethiopian census, the district had a total population of 103,852. Childbearing age women (CBAW) made up about 23,583(22.7%) of the total population. Administratively, the district was subdivided into 26 rural and 2 urban Kebeles/sub districts. The majority (91%) of the population lived in rural area and economically dependent on farming. The district is situated 350 meters above sea level and most of its parts were generally characterized by desert climatic condition with an annual temperature ranged between 26 and 38 degree Celsius [11]. Concerning health infrastructure the district had 22 health posts (HPs), 7 health centers (HCs) and one general hospital all providing maternal and child health care service. Moreover, it had 6 private clinics and 9 drug venders. According to the district health office 2011report, antenatal care and institutional delivery service were 67% and 43% respectively [12].

### Study design and Populations

A community based cross-sectional study that employed both quantitative and qualitative methods was undertaken. The source populations for the study were all women who gave birth at least once in the last two years preceding the survey irrespective of place and outcome of delivery. Study populations were randomly selected women of childbearing age who gave birth in the past two years prior to the survey on which the actual study was conducted. Mentally and/or physically incapable women, mothers who did not give birth at least once in the past two years prior to this survey and/or residing in the study area for less than six months were excluded from the study.

### Sample size and sampling procedure

A sample size of 485 was determined using sample size formula for estimating a single population proportion with the assumption that the proportion of institutional delivery service utilization as a determinant factor of knowledge about the danger signs of pregnancy and childbirth, margin of error, confidence interval, design effect and expected non response rate to be 18.4% [13], 5%, 95%, 2 and 5%, respectively. A total of four focus group discussions with an average number of 9 discussants per group were conducted on husbands and women who gave birth at least once. In-depth interviews were held with six health extension workers (community based primary health care providers).

Multistage sampling was used to select the study subjects. First, all the kebeles/sub-districts in the district were stratified into urban and rural. Then 1 out of 2 urban and 8 out of 24 rural Kebeles were randomly selected. The calculated sample size was proportionally allocated to urban (n = 55) and rural (n = 430) according to their number of households. Then, frames of households were prepared for each kebele in collaboration with the administrators of respective kebeles. Households with a woman who gave birth in the past 2 years prior to the survey were selected using systematic random sampling from the existing sampling frame of households. For selecting the study participants, different sampling intervals were used for each sub district. Whenever more than one eligible respondent was found in the same selected household, only one respondent was chosen by lottery method. For households with no eligible woman the immediate next household was selected and then subsequent households were selected according to the already pre-determined order. To supplement the quantitative data, four focus group discussions containing 18men and 18women discussants were organized. Information rich participants were selected through consultation with women association leaders and kebele administrators. Focus group homogeneity was assured by matching for age, sex, marital status and educational status. Six Health extension workers, who were assigned by the Ethiopian Ministry of health as community based primary health care providers were selected for in-depth interviews as they were more close to the community and had better awareness about knowledge and attitude of women about obstetric danger signs. Sample size for both focus group discussions and in-depth interviews was determined based on information saturation (selecting new focus group discussants stopped when generation of new ideas stopped).

### Data collection methods

Pretested and structured standard interviewer administered questionnaire, which was first prepared in English and translated into local language (Tigrigna) was employed to obtain information on socio-demographic, obstetric history, and knowledge of women about danger signs of pregnancy and childbirth. Nine 10^th^ grade completed female interviewers, who were fluent in the local language (Tigrigna) and were familiar with the local customs, collected the data. Two BSc health care workers (1 BSc Nurse & 1 health officer) with similar work experience were assigned to supervise the data collection process. Focus group discussions (FDGs) for husbands and in-depth interviews were conducted by the principal investigator and one registered BSc Nurse who speaks the local language. Focus group discussions for mothers were moderated by registered female BSc Nurse. A two day training complemented with practical exercise was given to data collectors and supervisors before the actual data collection regarding the aim of the study, data collection tool and procedures going through the questionnaires question by question.

### Data processing and analysis

Data were coded, entered and cleaned using EPI-INFO version 3.2 statistical packages and further cleaned and analyzed using SPSS for Windows version 16.0. Analysis of quantitative data was done sequentially starting with Univariate using descriptive techniques then bivariate analysis and finally a multivariate analysis was performed using SPSS version 16 statistical software to control for potential confounding factors. Statistical tests such as chi-square tests, odds ratio with 95% confidence interval were employed as appropriate. Data from the focus group discussions were transcribed and translated to English and categorized accordingly to main thematic areas manually. The findings were presented in the narratives in triangulation with the quantitative results.

#### Measurement

The danger signs are signs and symptoms of obstetric complications which occur during pregnancy, childbirth and immediately after delivery. Minor danger signs are obstetric complications which indicate minor obstetric complications. Whereas, major danger signs are complications which can easily be recognized and are signs of serious complications. They are grouped under phases of pregnancy, and childbirth. The major danger signs during pregnancy include severe vaginal bleeding, swollen hands/face and blurred vision. Whereas, key danger signs during childbirths are severe vaginal bleeding, prolonged labor (labor lasting more than 12 hours), convulsions and retained placenta [7].

Knowledge of women about obstetric danger signs were measured by the total number of correct spontaneous answers to 10 items on knowledge of pregnancy danger signs and 10 items on knowledge of labor and childbirth danger signs with a minimum score of 0 and maximum of 10. Spontaneous knowledge refers to the respondent's naming a sign without being asked about that sign by name. Only true obstetric complications spontaneously mentioned by individual respondents were included.

Accordingly, two categories were developed for each pregnancy and childbirth danger signs.

Had knowledge about pregnancy danger signs: Women who spontaneously mentioned at least two danger signs of Pregnancy.

Had no knowledge about pregnancy danger signs: Women who did not spontaneously mention two danger signs of Pregnancy.

Had knowledge about the danger signs of labor and childbirth: Women who spontaneously mentioned at least two danger signs of labor and childbirth.

Had no knowledge about the danger signs of labor and delivery: Women who did not spontaneously mention two danger signs of labor and childbirth.

### Ethical consideration

Ethical clearance was obtained from the institutional Review Board (IRB) of Addis Ababa University, college of health sciences. Official letter was written from Addis Ababa University (AAU), Department of Nursing and Midwifery to Tsegedie district. Informed consent was obtained from each respondent after a thorough explanation of the purpose and the procedures of the study. All responses were kept confidential and anonymous. Since majority of the respondents were illiterate we sought verbal consent but the consent form and information sheet were properly written and read by data collectors for the study subjects then when the study subjects agree on the content form they continued to ask if not they terminated and continued to other study subjects. In case of the age less than 18 years consent was obtained from caretakers. In general consent form was written but for the illiterate once it was read by the data collectors since they cannot read and sign. Oral consent is allowed by the institutional review board as long as the consent form and information sheet are properly written and read thoroughly to the illiterate participants.

## Results

### 4.1.1. Socio-demographic profile of respondents

A total of 485 women who had at least one birth prior to this survey were interviewed making a response rate of 100%. Two hundred nineteen (45.2%) were in the age group of 35 years and above with a mean age of 32.8±5.1 standard deviation (SD). The majority of the respondents accounting for 430 (88.7%) were rural dwellers, 362 (74.6%) currently in marital union, 424 (87.4%) Tigray by ethnicity, 462 (95.3%) orthodox, and 202 (41.6%) were housewives. Regarding their educational status, 264 (54.4) of the study subjects and 221 (57.6%) of their husbands were illiterate. Occupationally, 280 (72.9%) of husbands were farmers followed by merchants 68(17.7%). Out of the total respondents, all 485 (100%) volunteered to disclose their income and out of which 199 (41%) had a monthly income of 409–693 Ethiopian Birr (18 Ethiopian Birr equals to one American dollar). The mean number of people living in a household was 5.2±1.6 SD and 278(57.3%) households owned radio/television in their home [table 1].

**Table 1:** Socio-demographic characteristics of mothers aged 15–49 years in Tsegedie district, Tigray regional state, Northwest Ethiopia, June 2013 (n = 485).

Category	Subcategory	Frequency (%)
Place of residence	Urban	55 (11.3)
	Rural	430 (88.7)
Age at interview (in years)	15–19	8 (1.6)
	20–24	15 (3.1)
	25–29	103 (21.2)
	30–34	140 (28.9)
	> = 35	219 (45.2)
Marital Status of respondents	Single	24 (4.9)
	Married	362 (74.6)
	Divorced	50 (10.3)
	Widowed	27 (5.6)
	Separated	22 (4.5)
Religion of respondents	Orthodox	462 (95.3)
	Muslim	14 (2.9)
	Others*	9 (1.9)
Ethnicity of respondents	Tigray	424 (87.4)
	Amara	44 (9.1)
	Others **	17 (3.5)
Educational Status	Unable to read & write	264 (54.4)
	Able to read & write	93 (19.2)
	Primary education	58 (12)
	Secondary education	59(12.2)
	12+	11(2.3)
Occupation of respondents	Housewife	202 (41.6)
	Farmer	75(15.5)
	Employed	22(4.5)
	Merchant	52(10.7)
	Daily Laborer	124(25.6)
	Others***	10(2.1)
Have radio/TV	Yes	278(57.3)
	No	207(42.7)
Time taken to nearby health facility	<1 hour	239(49.3)
	> = 1 hour	246(50.7)
House hold income	150–408	92(19)
	409–693	199 (41)
	694–987	137 (28.2)
	> = 988	57 (11.8)

* Protestant, Catholic;

** Guragie, Oromo;

*** carpenter, house renting, student.

### 4.1.2. Past Obstetric characteristics of respondents

Majority 331 (68.2%) of the respondents were married at the age range of 15–19 years. Only ninety eight (20.2%) respondents had their first pregnancy before the age of 20 years and the mean age at first pregnancy was 21.5±2.76 SD. Regarding their obstetric history, majority 298 (61.4%) were gravida two to four and the average gravidity and parity were 3.4±1.6 SD and 3.34±1.6SD respectively. Regarding maternal health service utilization, 264 (54.4%) of the participants had at least one antenatal visit during their last pregnancy. Among those who attended ANC, only 43 (16.3%) have made 4 and more visits. Of those who said they have received health education during their last ANC visit 201 (74.2%) were informed about the danger signs related to pregnancy and childbirth, and 220 (81.2%) about the place of delivery [table 2].

**Table 2:** Past Obstetric characteristics of mothers aged 15–49 years in Tsegedie district, Tigray region, Northwest Ethiopia, June 2013.

Category	Subcategory	Frequency (%)
Age at first pregnancy (n = 485)	<20	98 (20.2)
	20–29	378 (77.9)
	30 & above	9 (1.9)
Parity (n = 485)	1	55 (11.3)
	2–4	302 (62.3)
	5 &above	128 (26.4)
ANC visit during last pregnancy (n = 485)	Yes	264 (54.4)
	No	221 (45.6)
Number of ANC visit during last pregnancy (n = 264)	1	34 (12.9)
	2–3	187 (70.8)
	4 & above	43 (16.3)
Place of last delivery (n = 485)	Health facility	153 (31.5)
	Home	332 (68.5)
Final decision maker on place of delivery (n = 485)	Self	133 (27.4)
	Family	129 (26.6)
	Relatives	223 (46)

### 4.1.3. Knowledge about danger signs of pregnancy and childbirth

Knowledge of respondents about delivery services was assessed by questions of danger signs related to pregnancy and childbirth. When the participants were asked to mention danger signs during pregnancy, the most common spontaneously mentioned danger signs were vaginal bleeding by 238 (49.1%), swelling of the legs or face by 202 (41.6%), and absence of fetal movement by 159 (32.8%). Two hundred eighty five (58.8%) respondents mentioned at least two danger signs during pregnancy and 170 (35.1%) didn't know any danger signs of pregnancy [table 3].

**Table 3:** Knowledge of the danger signs of pregnancy among mothers aged 15–49 years in Tsegedie district, Tigray region, Northwest Ethiopia, June 2013(n = 485).

Category 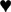	Frequency (%)
Severe Vaginal bleeding	238 (49.1)
Swelling of leg/face	202 (41.6)
Reduced/absence of fetal movement	159 (32.8)
Severe headache	140 (28.9)
Increased blood pressure	113 (23.3)
Persistent nausea and vomiting	106 (21.9)
Severe abdominal cramps	92 (19)
High Fever	87 (17.9)
Leakage of amniotic fluid without labor	74 (15.3)
Blurring of vision	26 (5.4)

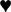
Multiple responses were possible.

*Almost all focus group discussants and in-depth interview participants reported that even though a significant progress has been registered since the last few years, most mothers were not educated and not familiar with most of the danger signs. Vaginal bleeding and decreased fetal movement were the most common obstetric complication mentioned as the danger signs of pregnancy.*

*The majority of women gave birth at home for the reason that they did not know obstetric danger signs; labor was considered easy and convenient and they believed that their husbands and other family members were just at home closely with them to provide any possible care in the way they felt right. These all made mothers comfortable to deliver at home.*

Similarly, the most commonly mentioned danger signs of labor and childbirth were excessive vaginal bleeding by 256 (52.8%), mal-presentation by171 (35.5%), labor lasting more than 12 hours by 151 (31.1%) and retained placenta by 146 (30.1%). Two hundred ninety nine (61.6%) of respondents mentioned at least two danger signs during labor and childbirth. One hundred fifty four (31.8%) didn't know any danger signs of labor and delivery [table 4].

**Table 4:** Knowledge of the danger signs of labor and childbirth among mothers aged 15–49 years in Tsegedie district, Tigray region, North West Ethiopia, June 2013(n = 485).

Category 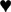	Frequency (%)
Vaginal bleeding	256 (52.8)
Mal-presentation/position	171 (35.3)
Prolonged labor (>12 hrs)	151 (31.1)
Retained Placenta (>1 hr)	146 (30.1)
Severe continuous abdominal pain	130 (26.8)
High fever	129 (26.6)
Increased blood pressure	121(24.9)
Convulsion/loss of consciousness	116 (23.9)
Cessation of labor pain	73 (15.1)
Sever head ache	47 (9.7)

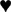
Multiple responses were possible.

*Danger signs of labor and childbirth mentioned by most of the focus group discussants and in-depth interview participants were retained placenta, bleeding and labor lasting more than a day.*

*Almost all participants stated that mothers in Tsegedie district were not even treated as human beings by their husbands. All house hold activities including the place where the mother should give birth were decided by the husband and the mother knew that the decision made by her husband was ultimate and accepted it. They did not have an opportunity for health care information and to know obstetric danger signs. These were the main reasons why women in Tsegedie district gave birth at home.*

### Maternal health service availability and accessibility factors

Almost all 482(99.4%) of the respondents reported that health facilities were available in their vicinity and 455 (93.8%) knew that delivery service was being provided in those facilities. Of those nearby health facilities, 404 (83.3%) were health centers, 46 (9.5%) were hospitals and 35 (7.2%) were health posts. For those who reported they had nearby health facilities, 246(50.7%) said the time taken on foot to the nearby health facility was one hour and above. Three hundred seventy one (76.5%) of the participants reported that transport service was available from their home to the nearby health facility. About three -fourth 125 (25.8%) of the respondents expressed that they had ever received delivery service in their nearby health facility and only 11 (2.3%) participants were charged for the service provided.

### Factors associated with knowledge of women on danger signs of pregnancy and childbirth

In a multivariate logistic regression analysis, educational status of the mother, possession of radio/television and place of delivery were independently associated with the mentioning of at least two danger signs of pregnancy and child birth. Whereas, age at interview showed an independent association only with knowledge of women about the danger signs of pregnancy (table 5).

**Table 5:** Association between knowledge of at least 2 danger signs of pregnancy and childbirth and selected socio-demographic and obstetric characteristics of mothers aged 15–49 years in Tsegedie district, Tigray region, Northwest Ethiopia, June 2013.

Category	Subcategory	AOR♣(95% CI) about danger signs of pregnancy	AOR♣(95% CI) for knowledge of women about danger signs of labor
Place of residence	Rural	1.00	1.00
	Urban	0.56(0.19,1.66)	0.60 (0.20,1.82)
Age at interview	15–24	1.00	1.00
	25–29	0.64(0.13,1.66)	1.80 (0.40,8.05)
	30–34	0.79(0.32,1.90)	3.02 (0.64,14.2)
	> = 35	**2.51(1.10,5.73)** *	1.82 (0.38,8.64)
Current Marital Status	Married	0.94(0.42,2.09)	1.17 (0.53,2.58)
	Not married	1.00	1.00
Educational Status	formal education	**2.23(1.53,2.88)** *	**3.92 (1.39,2.18)** *
	No formal education	1.00	1.00
Occupational status	Housewife	1.00	1.00
	Others↑	0.80(0.41,1.57)	0.92 (0.47,1.82)
Monthly income	>500 Birr	1.01(0.48,2.14)	0.86 (0.40,1.83)
	< = 500 Birr	1.00	1.00
Have radio/TV	No	1.00	1.00
	Yes	**2.41(1.22,4.76)** *	**2.04 (1.02,4.08)** *
Travel time to health facility	<1 hour	1.19(0.61,2.32)	1.52 (0.78,2.97)
	> = 1 hour	1.00	1.00
Parity	1	0.43(0.12,1.61)	0.43 (0.12,1.56)
	2–4	0.56(0.24,1.30)	0.80 (0.34,1.86)
	5 &above	1.00	1.00
ANC visits during last pregnancy	No	1.00	1.00
	Yes	4.99(0.69,35.99)	1.83 (0.17,20.33)
Number of ANC visit during last pregnancy	1	1.00	1.00
	2–3	2.04(0.81,5.15)	3.12 (1.24,7.87)
	4 & above	1.69(0.52,5.52)	1.70 (0.52,5.52)
Place of last delivery	Home	1.00	1.00
	Health facility	**11.87(4.74,29.73)** **	**12.2 (4.70,31.9)** **

↑ Farmer, Employed, Daily laborer, Carpenter, Student, House renting;

* significant at p<0.05;

** significant at p<0.01.

♣ Adjusted odds ratio.

Level of education showed strong statistical association with the mentioning of at least two danger signs during pregnancy and childbirth. Mothers with formal educational were about 2 times (AOR = 2. 23, 95%CI: [1.53, 2.88]) and 4times (AOR = 3. 92, 95%CI: [1.39, 2.18]) more likely to have higher knowledge of danger signs of pregnancy and childbirth as compared to their counterparts. Similarly, as respondents' age increased the likelihood of being knowledgeable about the danger signs of pregnancy increased (AOR = 2.51, 95%CI: [1.10, 5.73]).

The other strong predictor of knowledge about the danger signs of pregnancy and childbirth was place of delivery. Mothers who previously gave birth in health institutions were about12 times (AOR = 11. 87,95%CI: [4.74,29.73]; (AOR = 12. 2,95%CI: [4.70,31.9]) more likely to be knowledgeable about the danger signs of pregnancy and childbirth as compared to those who gave birth at home. Having a functional radio and/or television has also revealed a significant association with the mentioning of at least two danger signs of pregnancy (AOR = 2. 41, 95%CI: [1.22, 4.76]) and childbirth (AOR = 2. 04, 95%CI: [1.02, 4.08]).

## Discussion

This community based cross-sectional study identified factors that influence knowledge about the danger signs of pregnancy and childbirth among mothers who gave birth in the past two years prior to this survey in both urban and rural areas of Tsegedie District.

Inconsistent with a study done in rural Tanzania, India and rural Uganda [14], [15], [16], this study indicated that about half (49.1%) of the study subjects mentioned vaginal bleeding as danger sign of pregnancy. The Majority of the Focus group discussants (FGD) and in-depth interview participants had similar reports. But this is higher than the finding in Burkina Faso (39.4%), Guatemala (31.0%), Rewa district of Madhya Pradesh and Adigrat, Ethiopia (10.9%), [17], [18], [19], [20]. This difference might be due to socio-cultural difference and difference in the implementation of relevant health intervention programs. This could also be attributable to the time gap that since 2004 there could be improved in access and utilization of the health care information being provided. The finding of the present study also indicated that 285 (58.8%) of the respondents were knowledgeable about the danger signs of pregnancy. This finding is consistent with previous evidence from Kwazulu-Natal, province in South Africa [21]. However, it was lower than the report from India (79.2%) [22]. On the other hand, about one third (35.1%) of the respondents were unable to mention any danger sign of pregnancy, which is nearly consistent with the findings from Aleta Wondo, Ethiopia (39.0%) [9].

Level of education showed strong statistical association with the mentioning of at least two danger signs of pregnancy. Mothers with formal education were about 2 times (AOR = 2. 23, 95%CI: [1.53, 2.88]) more likely to have higher knowledge of danger signs of pregnancy as compared to their counterparts. This is comparable with reports from KwaZulu-Natal, Tanzania, Alexandria, Egypt, and Jordan [21], [23], [24], [25] and ***most FGD discussants and in-depth interview participants***. The possible explanations might be educated women have better access to health service information, improved perceptions of the causes of disease and treatment and can utilize such information optimally. In addition, educated women have greater autonomy to make decisions and have greater ability to use quality health care services. Similarly, a statistically significant difference was observed between the level of awareness and study subjects' age. In line with reports from Tanzania and Egypt [23], [24], respondents with higher age level were 2.5 times more likely to have higher knowledge about the danger signs of pregnancy (AOR = 2. 51, 95%CI: [1.10, 5.73]). This can be explained by the fact that increased awareness among older women may be related to their own experiences of pregnancy and delivery which is an important source of their information, especially those who had complications associated with their pregnancy. The other strong predictor of knowledge of women about the danger signs of pregnancy was a place of last delivery. The likelihood of being knowledgeable was 12 times higher when Mothers gave birth in health institutions, which is consistent with the study finding from Tanzania [23].

Key danger signs assessed during labor and childbirth included severe vaginal bleeding, prolonged labor, convulsions, and retained placenta. In line with the findings from Tanzania and India [13], [14], this study indicated that about half (52.8%) of the study subjects mentioned vaginal bleeding as a danger sign of child birth. Only 299 (61.6%) of the respondents mentioned at least two danger signs of labor and childbirth. This is higher than the report from Jordan (14.2%), and Aleta Wondo (40.9%) [25], [26].

Having formal education increased the likelihood of being knowledgeable about danger signs of labor and child birth by four times in this study, which is in line with studies done in Tanzania and Egypt [23], [24].The other strong predictor of knowledge of women about danger signs of labor and childbirth was place of last delivery. Similar with other study conducted in Tanzania [24], mothers who previously gave birth in health institutions were about12 times (AOR = 12. 2, 95%CI: [4.70, 31.9]) more likely to have higher knowledge about the danger signs of labor and delivery than those who gave birth at home.

The result of the present study also revealed that mass media exposure has statistically significant association with mentioning at least two danger signs of pregnancy and childbirth. The likelihood of being knowledgeable about the danger signs of pregnancy, labor and childbirth was two times higher for mothers who had functional Radio and/or Television. This is in agreement with a study done in Sekela district, North West Ethiopia and Nigeria [27], [28], which stated that educated women were more likely to utilize an institutional delivery service compared with uneducated women; this may be partly because they were better informed about obstetric complications and made better choices. This can further be explained by the fact that mass media is effective in information dissemination, which increases awareness about health care information and healthcare facilities that are available and fosters inter-personal communication, which could facilitate behavioral changes.

When interpreting the finding of this study, readers shall take into consideration the following limitations. First, the cross sectional nature of the data had made it impossible to reach at the causal relation between the different independent variables and knowledge of women about obstetric danger signs. Second, the source of data for this study was based on the self-report of respondents, and provided no validation of obtaining information with any objective source such as health facility cards. But it is logical to assume that biases are less likely in delivery related events as compared to sensitive issues such as sexual behavior and drug abuse, and respondents were informed about the importance of giving accurate responses and also assured the confidentiality of their responses. Third, this study was based on a survey of women that was conducted up to two years after the birth of a child. There may be women that died after giving birth but would not make it as part of the study because they were deceased by the time the interview would take place. The study might therefore miss these potential cases. Finally, there could be recalled bias since women were asked for events already happened within the last 2 years prior to the survey though the most recent births were considered.

## Conclusion

In this study a significant proportion of mothers were not knowledgeable about the danger signs of pregnancy, labor and childbirth. This indicates that many mothers are more likely to delay in deciding to seek care. Sever Vaginal bleeding was the most common spontaneously mentioned danger signs of pregnancy, labor and childbirth. Educational status of the mother, place of delivery and having functional Radio were found to be independent predictors of knowledge of women about the danger signs of pregnancy and childbirth.

Therefore, the FMOH, regional health bureau, zonal health department, Woreda health office as well as other partner organizations working in the areas of reproductive health should strengthen and scale up existing strategies including provision of information, education and communication targeting women, family, and the general community to promote the knowledge of reproductive age women about obstetric danger signs and hence can help Ethiopia achieve Millennium Development Goal five (MDG5) on a date.
